# Association between Bisphenol A and Prostate-Specific Antigen (PSA) among U.S. Older Males: National Health and Nutrition Examination Survey (NHANES), 2003–2012

**DOI:** 10.3390/nu16162589

**Published:** 2024-08-06

**Authors:** Qingyuan Li, Shipeng Gao, Youxian Zhang, Zhanpeng Xie, Lu Wang, Yu Li, Qiang Niu, Haiyan Li, Heng Guo, Rulin Ma, Jia He

**Affiliations:** 1 Medical School, Shihezi University, Shihezi 832003, China; lllqy_917@163.com (Q.L.); gspshihezi@163.com (S.G.); 13956220312@163.com (Y.Z.); xiezhanpeng2002@163.com (Z.X.); qq3480534563@163.com (L.W.); liyu108@shzu.edu.cn (Y.L.); niuqiang@shzu.edu.cn (Q.N.); lihaiyan@shzu.edu.cn (H.L.); guoheng@shzu.edu.cn (H.G.); marulin@shzu.edu.cn (R.M.); 2 Key Laboratory for Prevention and Control of Emerging Infectious Diseases and Public Health Security, Shihezi 832000, China; 3 Department of Preventive Medicine, School of Medicine, Shihezi University, Shihezi 832000, China

**Keywords:** bisphenol A, prostatic cancer, prostate-specific antigen, risk, NHANES

## Abstract

Background: There is growing evidence indicating that environmental endocrine disruptors may influence the development of prostate cancer. Despite this, the connection between BPA and PSA levels is still not fully understood and appears intricate. In this study, we aimed to assess the link between BPA exposure and PSA levels using data from the NHANES database. Methods: We conducted a weighted linear regression, logistic regression analysis, natural cubic spline (NCS), subgroup analysis, and interaction analysis on 2768 participants. Urinary BPA was considered the independent variable, while PSA was the dependent variable. Results: In the study, the average age of the participants selected was 62.70 years (±12.93). Age was negatively correlated with BPA, while PSA and BMI were positively correlated with BPA concentration (all of the *p*-value < 0.05). In the fully adjusted model, the weighted linear and logistic regression results showed that BPA was positively correlated with PSA and prostate cancer. NCS analysis results show that BPA and PSA have a non-linear relationship. Sensitivity and subgroup analyses showed similar results. In addition, there were interactions between BPA and age, PIR, education, HbA1c, high-density lipoprotein, smoking status, and Diabetes. Conclusions: There was a positive correlation between urinary BPA and PSA in older American males, especially when the BPA concentration was higher than 4.46 ng/mL. In future practical applications of prostate cancer screening, it is crucial to focus on individuals aged 75 years and older, as well as those with a PIR between 0 and 1, non-Hispanic black, and other risk groups to provide reference values for the primary and secondary prevention of prostate cancer.

## 1. Introduction

In 2020, 191,930 new cases of prostate cancer were diagnosed in the United States, accounting for more than 21% of new cancer cases in males and 10.4% of male cancer deaths in the United States. It was the second leading cause of death among male cancer patients [1]. Many factors contribute to prostate cancer, including exogenous factors such as smoking, alcohol consumption, diabetes, sexual behavior, chronic inflammation, and low ultraviolet exposure [2]. Research showed that environmental endocrine disruptors play a vital role in the development of reproductive system diseases such as prostate cancer [3]. Among them, urinary bisphenol A (BPA) is used daily. Significant sources of exposure to BPA include food packaging and dust, dental materials, medical devices, thermal paper, toys, and children’s and baby products [4]. The human prostate simultaneously expresses estrogen receptor-α (ER-α) [5] during development, so inactivating mutations of androgen receptors and the abnormal biosynthesis of these receptors are associated with a lack of or immature male prostate development. Research shows that BPA interacts directly with ER-alpha and, through this effect, can promote the growth of prostate cancer [6].

However, one study showed that low concentrations of BPA stimulated the proliferation of androgen receptor-expressing cells, which inhibited the growth of prostate cancer cells [7]. In contrast, another study from Australia found no significant association [8]. At the same time, experiments based on mice show that exposure to BPA alone at any dose will not cause prostate lesions [9]. Therefore, the association between prostate cancer and BPA exposure remains controversial.

Detecting serum prostate-specific antigen (PSA) concentration is vital in early prostate cancer screening [10]. The results of a randomized trial supported that PSA screening can moderately reduce prostate cancer mortality in 1000 males. Adequate evidence from randomized clinical trials shows that PSA-based screening programs in men aged 55 to 69 years may prevent approximately 1.3 deaths from prostate cancer over approximately 13 years per 1000 men screened [11]. Therefore, exploring PSA levels in people exposed to BPA is essential to studying the relationship between BPA exposure and prostate cancer.

NHANES has nationally representative chronic disease samples and comprehensive data [12], providing us with high-quality, representative samples. This study analyzed data drawn from the National Health and Nutrition Examination Survey (NHANES) from 2003 to 2012, focusing on older men in the United States. Statistical methods such as weighted linear regression, weighted logistic regression analysis, natural cubic spline (NCS), and a subgroup analysis were used to explore the association between BPA exposure and prostate cancer biomarkers (such as PSA). These efforts provide public health reference values for early screening and the timely intervention of prostate cancer.

## 2. Materials and Method

### 2.1. Data Availability

NHANES was a nationally representative survey of the noninstitutionalized U.S. civilian population conducted by the Centers for Disease Control and Prevention (CDC)’s National Center for Health Statistics (NCHS). We used data from 2003 through to publicly available data on the participants recruited during 2012; the National Health Statistics Research Ethics Review Board approved the 2003–2012 NHANES surveys. All participants consented to the NHANES survey.

### 2.2. Study Population

**Figure 1:**
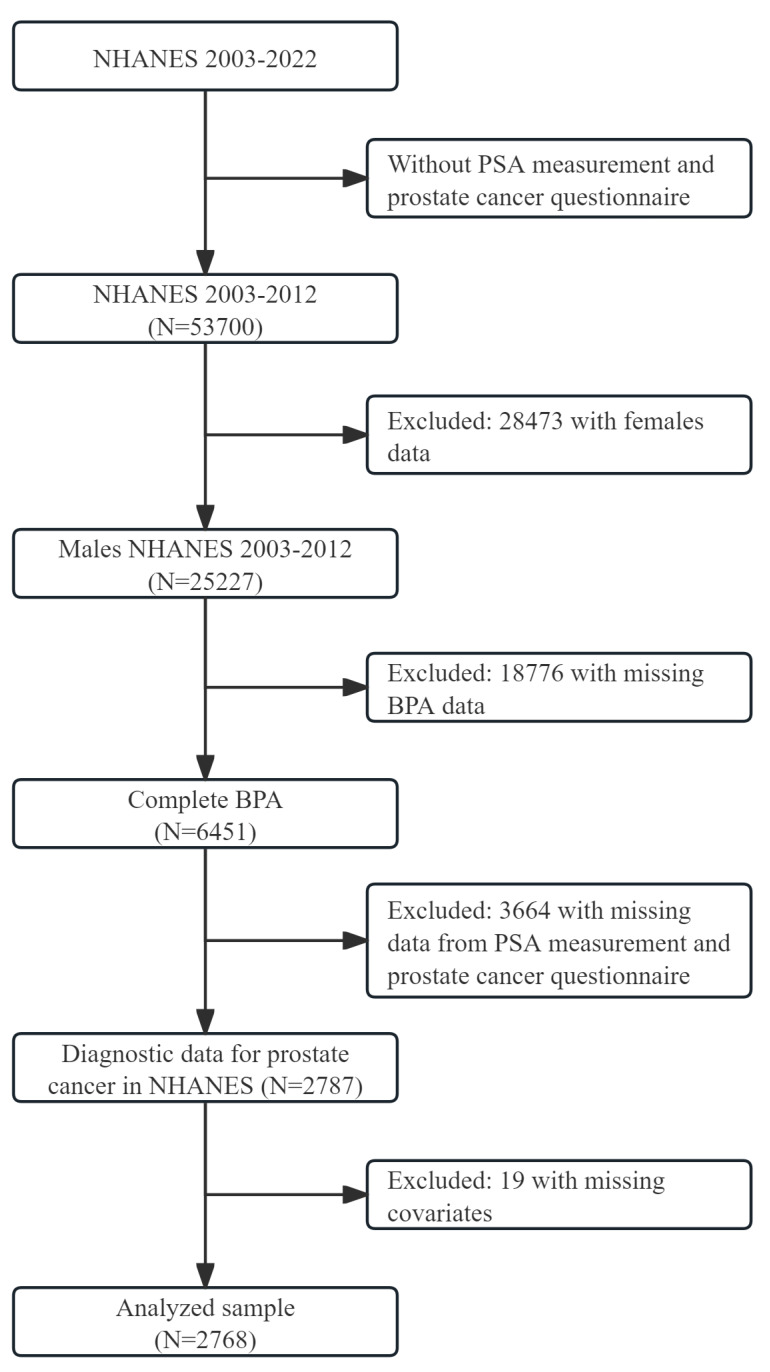
Flowchart for selecting the study’s participants.

Our study included two years of data from four cycles of the NHANES survey of people aged 20 and older from 2003 to 2012. These data included the participants’ PSA concentration, urinary BPA concentration, sociodemographic data, laboratory data, and medical examination data. Ineligible participants were excluded based on the following exclusion criteria: (1) female participants; (2) missing PSA; (3) missing urinary BPA concentration; (4) missing covariates. 2768 out of the 53,700 participants were eligible for our study after screening (Figure 1). Our study adhered to the World Medical Association’s Declaration of Helsinki during design and conduct.

In our study, urinary BPA was the target independent variable downloaded from the NHANES website. Collaborative laboratory services regularly refine these laboratory methods. More details on the test rationale and clinical relevance can be found on the NHANES website and in other articles [13]. A set PSA concentration (ng/mL) was the dependent variable and the study included weighted linear regression, weighted logistic regression analysis, natural cubic spline (NCS), and other statistical methods for theanalyses. The total PSA from the participants was was recorded by Beckman Access in the Division of Laboratory Medicine Immunology and the serum total PSA was recorded using the Hybritech PSA method. In this study, we used total serum PSA as the outcome variable related to BPA. We selected the following covariates based on previous articles on the link between prostate cancer and BPA [14]. Continuous variables included the participants’ age, poverty income ratio (PIR), body mass index (BMI), total cholesterol (mmol/L), high-density lipoprotein (mmol/L), and glycosylated haemoglobin (%); categorical variables included the participants’ race, education, marital status, smoking status, drinking status, history of hypertension, history of diabetes, and tumor history. For detailed information, please visit NHANES’s official website.

### 2.3. Statistical Analysis

According to the standards of the U.S. Centers for Disease Control and Prevention guidelines, we conducted statistical analyses on urinary BPA and PSA levels. Also, we divided the PSA concentrations into low cancer risk (PSA < 4 ng/mL) and high cancer risk (PSA ≥ 4 ng/mL) [15]. In both groups, for the results obtained using NHANES, the data were expressed as the mean with a 95% confidence interval (CI), the median with the interquartile range, or the prevalence of prostate cancer (%) expressed. We used the *t*-test and χ^2^ test to determine the differences between groups. Because urinary BPA concentrations were strongly right-skewed, the estimates of urinary BPA concentrations needed to be adjusted for urinary creatinine (Cr) (BPA/Cr, ng/mg) when treated as continuous variables. Then, weighted a linear regression, weighted logistic regression analysis, natural cubic spline (NCS), subgroup analysis, sensitivity analysis, and interaction were performed, adjusting for race/ethnicity, age, PIR, education, marital status, total cholesterol, HDL, glycohemoglobin, BMI, diabetes, hypertension, tumor history, smoking, and drinking status; the results were reported as OR and 95% CI. Because NHANES is a nationally representative survey using a complex, stratified, multistage, probabilistic cluster design, we applied a complex sample design using sampling weights to produce results that were representative of the U.S. population. The analyses were performed using combined sampling weights as per NHANES guidelines, and all analyses were performed using the R statistical package, with statistical significance assessed at a type I error rate of *p* < 0.05.

## 3. Results

### 3.1. Baseline Characteristics of Selected Participants Subsection

The weighted distribution of baseline characteristics is presented in Table 1, which includes sociodemographic data, laboratory data, personal life history from physical examinations, as well as dietary and comorbidity data for the participants selected from the NHANES surveys conducted between 2003 and 2012. In this study, the mean age of the selected participants was 62.70 years (±12.93). We classified the urinary BPA levels into four quartiles (Q1–Q4). The results indicated a downward trend in BMI with increasing urinary BPA concentration (*p*-value < 0.05). Additionally, non-Hispanic white people constituted the predominant demographic among the participants (*p* < 0.05).

### 3.2. The Connection between BPA Concentrations and PSA

The analysis results of the weighted linear model are presented in Table 2. A positive association was observed between urinary BPA and PSA concentrations in both unadjusted and adjusted models. Specifically, results from the fully adjusted model indicated that for each unit increase in BPA, the PSA concentration increased by 2.760 ng/mL (1.506, 4.014). To further investigate the relationship between BPA and PSA, BPA levels were divided into quartiles. In the fully adjusted model, compared to the Q1, each unit increase in BPA in the Q4 was associated with a PSA concentration increase of 1.074 ng/mL (0.862, 1.286). Additionally, we categorized the PSA concentrations into low cancer risk (PSA < 4 ng/mL) and high cancer risk (PSA ≥ 4 ng/mL). The results of the weighted logistic regression indicated that in the fully adjusted model, a one-unit increase in BPA was linked to a 42.5% increase in the likelihood of high cancer risk. When BPA was divided into quartiles, the fully adjusted model revealed that the risk of prostate cancer increased by 78.0% for each unit increase in BPA when comparing the Q4 to the Q1 (Table S1). Furthermore, results from the NCS indicated that both urinary BPA and PSA exhibited a non-linear relationship. However, when PSA was treated as a dichotomous variable, the non-linear relationship between urinary BPA and the prostate cancer risk was no longer evident after the model’s adjustment (Figure S1).

### 3.3. Stratified Associations between BPA Concentrations and PSA

**Figure 2:**
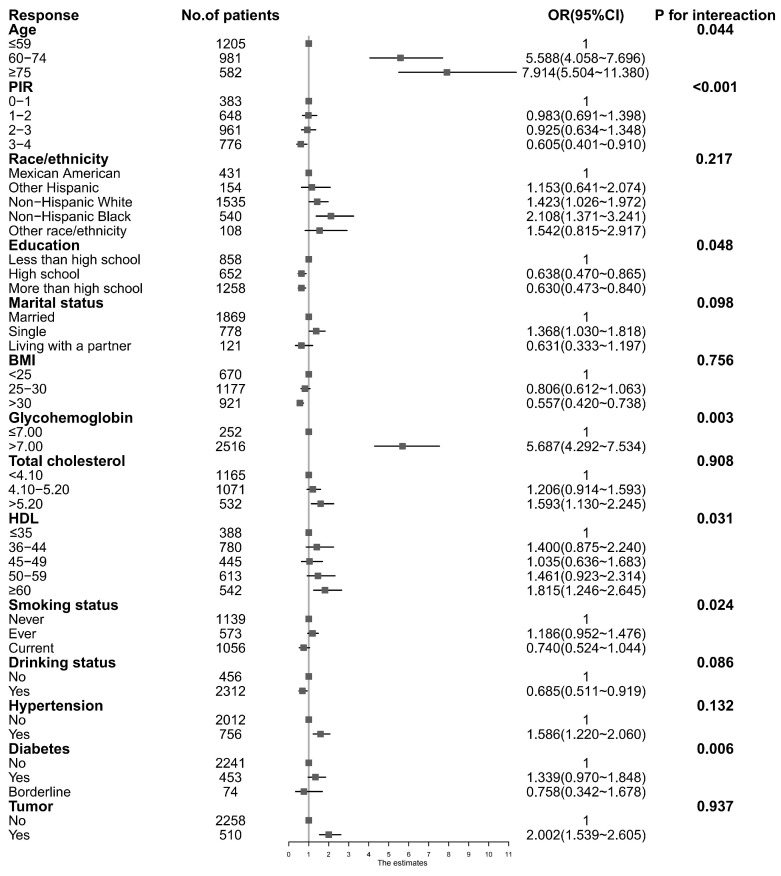
Subgroup analysis of urinary BPA and PSA. CI, confidence interval; OR, odds ratio.

As shown in Figure 2, we conducted a subgroup analysis based on factors such as age, PIR, etc., to further evaluate the relationship between urinary BPA and PSA. We used 4 ng/mL as the cutoff to divide PSA into low and high cancer risks. For the cancer risk in both groups, the results show that the risk factors were as follows: the age was ≥ 75 years old, the PIR was between 0 and 1, the ethnic group was non-Hispanic black, the education level was less than high school level, the relationship status was single, the BMI value was less than 25, the glycohemoglobin level was > 7.00%, the total cholesterol was > 4.10 mmol/L, and the high-density lipoprotein level was > 3.50 mmol/L. Patients who have hypertension and cancer have a higher risk of prostate cancer as the concentration of BPA increases. In addition, we found interactions between urinary BPA and age, PIR, education, glycohemoglobin, HDL, smoking status, and diabetes (*p* for interaction < 0.05).

### 3.4. Identification of Sensitivity Analysis

We conducted sensitivity analyses (Tables S2–S5) to confirm the accuracy and stability of the results. Based on previous data, we removed participants who were taking drugs to treat prostate cancer and those who had tumors. Based on this, we conducted weighted linear and logistic regression analyses. The results found that removing the drug-taking population was associated with the tumor population; the results were not much different from before (all of the OR > 1, all of the *p*-value < 0.05).

## 4. Discussion

This study represents the first investigation into the potential link between urinary BPA and PSA levels. The results indicated a positive correlation between urinary BPA concentrations and PSA levels, following a series of weighted linear and logistic regression analyses. Specifically, for each unit increase in urinary BPA (ng/mL), the PSA concentration rises by 2.760 ng/mL, correlating with a 42.5% increase in the prostate cancer risk. Furthermore, the NCS analysis revealed a non-linear relationship between urinary BPA and PSA. Notably, the significance of these results was heightened when urinary BPA concentrations exceeded 4.46 ng/mL. Our findings suggest that previous research has largely overlooked this area, indicating the potential utility of this cutoff value for early prostate cancer screening and the identification of additional at-risk populations. However, it is important to note that even with the application of a cutoff value, there remains a risk of overlooking some prostate cancer cases. Thus, in practical applications, it is crucial to combine this value with other indicators for a more comprehensive diagnosis. Additionally, we found that age, PIR, and other variables may interact with the urinary BPA concentration. We further validated the robustness of these findings through subgroup and sensitivity analyses.

The relationship between BPA and the risk of prostate cancer remains a subject of considerable controversy. PSA testing is commonly employed for the early screening of prostate cancer. Consequently, investigating the relationship between urinary BPA levels and PSA concentrations holds significant clinical implications for the prevention and treatment of early prostate cancer. However, there is a lack of reported studies examining the correlation between urinary BPA and PSA levels. This study supports a positive correlation between urinary BPA and PSA concentrations, which is particularly pronounced when urinary BPA levels exceed 4.46 ng/mL. Furthermore, numerous previous studies have identified estrogen as a key causative factor in the development of prostate cancer [9]. This may be why exposure to BPA increases the incidence of prostate cancer. BPA indirectly activates the androgen receptor mutation (AR-T877 A) through its interaction with ER-β. The mutant androgen receptor (T877 A) promotes cell growth in the LNCaP and LAPC 4 cell lines, significantly contributing to prostate growth and differentiation. Additionally, BPA may enhance the proliferation of human prostate cancer cells by activating the endogenous AR-T877 A mutation [16]. Because of the risk, further research is needed to explore the mechanisms by which BPA affects PSA concentrations and the implications for prostate cancer screening. Consistent with the research results of Mottet N, Ilaria Cimmino, Belluti S, et al. [2,4,5,6], a 2017 study from Hong Kong showed that exposure to high levels of BPA was positively associated with the risk of prostate cancer (OR = 1.57, 95% CI: 1.01–2.44) [17]; the OR value was larger than that obtained by the logistic regression in this article. This may be due to the different autoantibody profiles between different patients, which leads to a decrease in the sensitivity of PSA in detecting prostate cancer, resulting in false positives. Adverse conditions were related [18]. As we observed in this study that higher levels of BPA were positively associated with PSA (Figure S1), the risk of prostate cancer may be higher in those with high BPA levels. However, some studies on BPA exposure and prostate cancer risk still have conflicting conclusions.

This study’s results showed that there was no significant correlation between male urinary BPA concentration and reproductive health [19]. This study selected 169 male patients from Belgium who were <50 years old and had a BMI ≤ 35 kg/m^2^. Our study population had an average age of 62.70 ± 12.93 years old and an average BMI of 28.74 ± 5.81 kg/m^2^. In fact, after the age of 50, the risk of prostate cancer increases significantly [20], and another study pointed out that for every 5 kg/m^2^ increase in BMI, the risk of localized prostate cancer was reduced by 6% (RR = 0.940, 95% CI: 0.910~0.907) [21]. At the same time, a study from South Korea showed that the BPA level in Korean adults’ urine increased with age. There was a positive correlation trend, which showed that the Korean adult population aged 60–69 has the highest BPA content [22]. This may be due to the fact that younger people have been exposed to BPA for a shorter time than older people, resulting in lower levels in younger age groups. The concentration of BPA was relatively low, resulting in a low risk of prostate cancer. Therefore, the race and age of the participants may be the reasons for the conflicting relationship between BPA and PSA [15]. At the same time, the results of animal model studies based on rats also indicate that exposure to BPA at any dose alone will not cause prostate lesions [23]. This may be due to the fact that most rat species, including the Sprague–Dawley used in this study, do not show spontaneous development of prostate cancer, which contributes to differences in the cancer risk between the two species as the use of rats as a model for prostate cancer requires potent chemical carcinogens or prolonged exposure to natural sex steroids with high receptor affinity. In this context, it was not surprising that BPA alone was not a carcinogen in rat prostates due to its reduced affinity for nuclear ERs in prostate cells relative to E2 [24]. This article and most studies support the positive correlation between urinary BPA and the risk of prostate cancer, which may be helpful for subsequent prostate cancer screening. However, since prostate cancer is currently the most common malignant tumor in the male reproductive system, and there is a lack of information, based on NHANES data, on the relationship between BPA and prostate cancer, studies are still needed to evaluate the relationship between BPA and levels of the prostate cancer tumor marker, PSA. In addition, Daniela Terracciano and others found that PSA density was more effective in screening prostate cancer than PSA [25], indicating that the study of PSA and prostate cancer still has essential research value. This study also suggests our future research directions. We need to conduct further research to analyze the association between BPA and PSA density to provide crucial public health reference values for primary and secondary prostate cancer prevention.

In addition, we also conducted a subgroup analysis study. The results showed that patients’ aged ≥ 75 years old, who have a household income ratio between 0 and 1, an ethnicity of non-Hispanic black, education of less than high school level, a relationship status of single, BMI value below 25, glycohemoglobin level > 7.00%, total cholesterol > 4.10 mmol/L, high-density lipoprotein level > 3.50 mmol/L, and patients who have hypertension and cancer should be our focus for screening BPA concentration and PSA. At the same time, age, PIR, education status, glycated hemoglobin, HDL-C, smoking history, and diabetes history may have interactive effects related to BPA concentration. One study showed an inverse correlation between BPA and BMI in patients aged ≤ 20 years, while a dose–response correlation existed between BPA and BMI in people aged 20 to 50 years [26]. Another study noted that serum triglycerides were independently and inversely associated with PSA in U.S. males, with higher triglyceride concentrations associated with lower PSA, so the likelihood of detecting asymptomatic prostate cancer may be lower in high-triglyceride populations. Moreover, for every unit increase in serum triglycerides (mg/dL), the PSA concentration decreases by 0.0043 ng/mL (−0.0082, −0.0005) [27], which may be the reason why people with lower BMI had a higher risk of prostate cancer. In summary, it was suggested that participants’ aged ≥ 75 years old, who have a PIR of 0–1, an ethnicity of non-Hispanic black, an education level of less than high school level, a glycohemoglobin level ≥ 7.00%, total cholesterol > 5.20 mmol/L, HDL ≤ 35 mmol/L, who are non-daily smokers, and who have diabetes may be at risk for prostate cancer. In practical applications, we should focus on the BPA concentration of these groups to make precision medicine possible.

This study had several advantages compared with previously published articles. First, this study was the first NHANES-based study on the relationship between urinary BPA and PSA. Secondly, we performed a sensitivity analysis by removing the medication population and the population confirmed to have prostate cancer, considering and evaluating the impact of other factors that may affect the results, and improving the reliability of the research results. Finally, a subgroup analysis was performed based on the fully adjusted model to explore the impact of covariates on the results and improve the accuracy and stability of the research results. However, our study also had some limitations in interpreting the results. First, in our study, due to the inherent limitations of the NHANES database as a cross-sectional survey, it was not easy to distinguish cause and effect, so prospective cohort studies were needed to confirm. At the same time, although this study adjusted some variables, there were still variables that may affect the research results that were not included. Second, our survey was based on the NHANES database and was limited to people in the United States. Therefore, generalizability was geographically limited. The above points all require further evaluation and investigation in the future. In addition to this, although the early detection of prostate cancer uses screening with PSA and its free fraction widely, this situation was associated with a large number of males undergoing unnecessary prostate biopsies and an increase in testing for benign prostatic hyperplasia and those with a grade equal to 1, according to the ISUP group of indolent cancers [25]. Then, Meeker et al.’s research found that repeated urine samples collected from the same man weeks to months apart from one another were weakly correlated (r = 0.18). Thus, another limitation in the present study is the likelihood of an exposure measurement error due to the high within-individual temporal variability in BPA exposure and the availability of multiple BPA measures from only a subset of participants [28]. In contrast, the widespread use of PSA has led to a high overdiagnosis rate. Overdiagnosis leads to unnecessary definitive treatment of prostate cancer and produces harmful side effects [29]. Therefore, perhaps the use of a combined model, including a prostate health index and multiparametric magnetic resonance, may be more effective in identifying prostate cancer at the time of initial diagnosis.

## 5. Conclusions

This study posits a positive correlation between exposure to BPA and elevated PSA concentrations, particularly when BPA levels exceed 4.46 ng/mL. In future practical applications of prostate cancer screening, it is crucial to focus on individuals aged 75 years and older, as well as those with a PIR between 0 and 1. Additionally, attention should be given to non-Hispanic black, individuals with an education level below high school, those with a glycohemoglobin level ≥ 7.00%, total cholesterol levels exceeding 5.20 mmol/L, HDL levels at or below 35 mmol/L, non-daily smokers, and patients diagnosed with diabetes. These efforts provide reference values for the primary and secondary prevention of prostate cancer.

## Figures and Tables

**Table 1:** Baseline characteristics of the selected participants.

Urinary BPA Concentrations (ng/mL)	Overall	Q1	Q2	Q3	Q4	*p*-Value
N	2768	703	712	667	686	
PSA ng/mL	3.21 ± 6.24	2.59 ± 3.53	2.80 ± 5.12	3.03 ± 6.72	4.46 ± 8.43	<0.001
Sociodemographic variables						
Age, mean ± SD (years)	62.70 ± 12.93	63.09 ± 12.60	62.64 ± 12.78	61.46 ± 13.14	63.59 ± 13.13	0.019
PIR, mean ± SD (years)	2.79 ± 1.55	2.80 ± 1.52	2.86 ± 1.54	2.81 ± 1.56	2.71 ± 1.56	0.348
Race/ethnicity, n (%)						0.006
Mexican American	431 (15.6)	128 (18.2)	101 (14.2)	97 (14.5)	105 (15.5)	
Other Hispanic	154 (5.6)	34 (4.8)	37 (5.2)	46 (6.9)	37 (5.4)	
Non-Hispanic white	1535 (55.5)	390 (55.5)	399 (56.0)	357 (53.5)	389 (56.7)	
Non-Hispanic black	540 (19.5)	115 (16.4)	135 (19.0)	152 (22.8)	138 (20.1)	
Other race/ethnicity	108 (3.9)	36 (5.1)	40 (5.6)	15 (2.2)	17 (2.5)	
Education, n (%)						0.658
Less than high school	858 (31.0)	229 (32.6)	217 (30.5)	199 (29.8)	213 (31.0)	
High school	652 (23.6)	158 (22.5)	174 (24.4)	168 (25.2)	152 (22.2)	
More than high school	1258 (45.4)	316 (45.0)	321 (45.1)	300 (45.0)	321 (46.8)	
Marital status, n (%)						0.101
Married	1869 (67.5)	496 (70.6)	482 (67.7)	452 (67.8)	439 (64.0)	
Single	778 (28.1)	176 (25.0)	203 (28.5)	187 (28.0)	212 (30.9)	
Living with a partner	121 (4.4)	31 (4.4)	27 (3.8)	28 (4.2)	35 (5.1)	
Medical examination and personal life history						
BMI, kg/m^2^	28.74 ± 5.81	28.24 ± 5.46	28.81 ± 5.67	29.20 ± 6.80	28.71 ± 5.20	0.033
Variables of laboratory data						
Glycohemoglobin (%)	5.90 ± 1.11	5.90 ± 1.16	5.92 ± 1.10	5.89 ± 1.12	5.91 ± 1.05	0.496
Total cholesterol, mmol/L	5.06 ± 1.10	5.13 ± 1.14	5.04 ± 1.09	5.01 ± 1.05	5.05 ± 1.13	0.325
High-density lipoprotein, mmol/L	1.28 ± 0.39	1.32 ± 0.42	1.27 ± 0.38	1.27 ± 0.39	1.25 ± 0.36	0.080
Comorbidities, n (%)						
Smoking status						0.241
Never	1056 (38.2)	247 (35.1)	275 (38.6)	265 (39.7)	269 (39.2)	
Current	573 (20.7)	151 (21.5)	148 (20.8)	143 (21.4)	131 (19.1)	
Ever	1139 (41.1)	305 (43.4)	289 (40.6)	259 (39.7)	286 (41.7)	
Drinking status	2312 (83.5)	580 (82.5)	593 (83.3)	561 (84.1)	578 (84.3)	0.904
Yes	2312 (83.5)	580 (82.5)	593 (83.3)	561 (84.1)	578 (84.3)	
No	456 (16.5)	123 (17.5)	119 (16.7)	106 (15.9)	108 (15.7)	
Hypertension						0.087
Yes	756 (27.3)	210 (29.9)	186 (26.1)	165 (24.7)	195 (28.4)	
No	2012 (72.7)	493 (70.1)	526 (79.6)	502 (75.3)	491 (71.6)	
Diabetes						0.287
Yes	453 (16.4)	116 (16.5)	121 (17.0)	102 (15.3)	114 (16.6)	
No	2241 (81.0)	566 (80.5)	567 (79.6)	553 (82.9)	555 (80.9)	
Borderline	74 (2.7)	21 (3.0)	24 (3.4)	12 (1.8)	17 (2.5)	

Q1–Q4: Grouped by quartile according to the urinary BPA concentrations. Our data included PSA concentrations, sociodemographic data, laboratory data, medical examination (personal life history), dietary, and comorbidities data for the second analysis.

**Table 2:** Weighted linear model of urinary BPA and PSA.

Exposure	Non-Adjusted Model			Minimally Adjusted Model			Fully Adjusted Model		
	Estimate	95% CI	*p*-Value	Estimate	95% CI	*p*-Value	Estimate	95% CI	*p*-Value
Urinary BPA	3.416	(2.007, 4.825)	0.018	2.706	(1.442, 3.974)	0.037	2.760	(1.506, 4.014)	0.034
Urinary BPA									
Q1	Ref.			Ref.			Ref.		
Q2	0.073	(−0.118, 0.265)	0.703	0.135	(−0.033, 0.304)	0.425	0.165	(−0.002, 0.332)	0.328
Q3	0.232	(0.028, 0.436)	0.259	0.413	(0.204, 0.622)	0.053	0.311	(0.235, 0.638)	0.036
Q4	1.070	(0.816, 1.324)	<0.001	1.025	(0.821, 1.228)	<0.001	1.074	(0.862, 1.286)	<0.001
*p* for trend			<0.001			<0.001			0.011

Non-adjusted model adjusts for nothing. Minimally adjusted model adjusts for race/ethnicity, age, and PIR. Fully adjusted model adjusts for race/ethnicity, age, PIR, education, marital status, total cholesterol, HDL, glycohemoglobin, BMI, diabetes, hypertension, tumor history, smoke status, and drinking status.

## Data Availability

All data are available at NHANES website https://www.cdc.gov/nchs/nhanes/index.htm (accessed on 20 August 2022).

## Supplementary Materials

The following supporting information can be downloaded at https://www.mdpi.com/article/10.3390/nu16162589/s1. Table S1: weighted generalized logistic model of urinary BPA and PSA; Table S2: sensitivity analysis of the relationship between BPA and PSA, excluding patients taking prostate cancer drugs, when PSA is a binary variable; Table S3: sensitivity analysis of the relationship between BPA and PSA, excluding patients taking prostate cancer drugs, when PSA is a continuous variable; Table S4: sensitivity analysis of the relationship between BPA and PSA after excluding tumor patients when PSA is a binary variable; Table S5: sensitivity analysis of the relationship between BPA and PSA after excluding tumor patients when PSA is a continuous variable; Figure S1: the non-linear relationship between BPA and PSA.
