# Supplementary material for: Association between Bisphenol A and Prostate-Specific Antigen (PSA) among U.S. Older Males: National Health and Nutrition Examination Survey (NHANES), 2003–2012
Source: Nutrients. 2024 Aug 6;16(16):2589. doi: 10.3390/nu16162589 (PMC11357130; doi:10.3390/nu16162589)
Supplement: Supplementary file 1 [file nutrients-16-02589-s001.zip › nutrients-3128829-supplementary.pdf]

Table S1. weighted generalized logistic model of urinary BPA and PSA

Outcomes	OR with CI	*p*-value
overall		
Non-Adjusted Model	1.541(1.101,2.157)	0.013
Minimally Adjusted Model	1.423(1.058,1.913)	0.021
Fully Adjusted Model	1.425(1.065,1.906)	0.018
Q1		
Non-Adjusted Model	Ref.	
Minimally Adjusted Model	Ref.	
Fully Adjusted Model	Ref.	
Q2		
Non-Adjusted Model	0.953(0.743,1.221)	0.701
Minimally Adjusted Model	0.953(0.730,1.244)	0.722
Fully Adjusted Model	0.986(0.753,1.291)	0.917
Q3		
Non-Adjusted Model	0.995(0.774,1.279)	0.966
Minimally Adjusted Model	1.091(0.833,1.430)	0.527
Fully Adjusted Model	1.141(0.868,1.502)	0.345
Q4		
Non-Adjusted Model	1.671(1.319,2.116)	≤0.001
Minimally Adjusted Model	1.714(1.328,2.214)	≤0.001
Fully Adjusted Model	1.780(1.373,2.308)	≤0.001

Non-adjusted model adjusts for nothing. Minimally adjusted model adjusts for race/ethnicity, age, and PIR. Fully adjusted model adjusts for race/ethnicity, age, PIR, education, marital status, total cholesterol, HDL, glycohemoglobin, BMI, diabetes, hypertension, tumor history, smoke status, and drinking status.

Table S2. sensitivity analysis of the relationship between BPA and PSA, excluding patients taking prostate cancer drugs, when PSA is a binary variable

Outcomes	OR with CI	*p*-value
overall		
Non-Adjusted Model	1.519(1.091,2.115)	0.014
Minimally Adjusted Model	1.391(1.028,1.883)	0.033
Fully Adjusted Model	1.393(1.034,1.878)	0.030
Q1		
Non-Adjusted Model	Ref.	
Minimally Adjusted Model	Ref.	
Fully Adjusted Model	Ref.	
Q2		
Non-Adjusted Model	0.965(0.750,1.240)	0.778
Minimally Adjusted Model	1.032(0.776,1.371)	0.831
Fully Adjusted Model	1.146(0.849,1.546)	0.374
Q3		
Non-Adjusted Model	1.002(0.778,1.292)	0.986
Minimally Adjusted Model	1.154(0.865,1.540)	0.330
Fully Adjusted Model	1.281(0.946,1.737)	0.109
Q4		
Non-Adjusted Model	1.632(1.285,2.072)	≤0.001
Minimally Adjusted Model	1.716(1.307,2.253)	≤0.001
Fully Adjusted Model	1.936(1.452,2.582)	≤0.001

Non-adjusted model adjusts for nothing. Minimally adjusted model adjusts for race/ethnicity, age, and PIR. Fully adjusted model adjusts for race/ethnicity, age, PIR, education, marital status, total cholesterol, HDL, glycohemoglobin, BMI, diabetes, hypertension, tumor history, smoke status, and drinking status.

Table S3. sensitivity analysis of the relationship between BPA and PSA, excluding patients taking prostate cancer drugs, when PSA is a continuous variable

Exposure	Non-Adjusted Mode			Minimally Adjusted Model			Fully Adjusted Model		
	Estimate	95%CI	P-value	Estimate	95%CI	P-value	Estimate	95%CI	P-value
Urinary BPA	3.262	（1.896,4.628）	0.020	2.580	(1.365,3.795)	0.038	2.581	(1.386,3.776)	0.037
Urinary BPA									
Q1		Ref.			Ref.			Ref.	
Q2	0.075	(-0.120,0.271)	0.702	0.215	(0.046,0.383)	0.207	0.204	(0.055,0.354)	0.180
Q3	0.162	(-0.023,0.347)	0.385	0.382	(0.187,0.577)	0.055	0.330	(0.157,0.502)	0.063
Q4	1.030	(0.7648,1.296)	<0.001	0.952	(0.748,1.156)	<0.001	0.985	(0.794,1.177)	<0.001
p for trend			<0.001			<0.001			0.004

Non-adjusted model adjusts for nothing. Minimally adjusted model adjusts for race/ethnicity, age, and PIR. Fully adjusted model adjusts for race/ethnicity, age, PIR, education, marital status, total cholesterol, HDL, glycohemoglobin, BMI, diabetes, hypertension, tumor history, smoke status, and drinking status.

Table S4. sensitivity analysis of the relationship between BPA and PSA after excluding tumor patients when PSA is a binary variable

Outcomes	OR with CI	*p*-value
overall		
Non-Adjusted Model	1.521(1.020,2.267)	0.040
Minimally Adjusted Model	1.365(0.943,1.974)	0.098
Fully Adjusted Model	1.359(0.951,1.941)	0.090
Q1		
Non-Adjusted Model	Ref.	
Minimally Adjusted Model	Ref.	
Fully Adjusted Model	Ref.	
Q2		
Non-Adjusted Model	0.923(0.696,1.223)	0.576
Minimally Adjusted Model	0.946(0.686,1.306)	0.737
Fully Adjusted Model	1.020(0.726,1.434)	0.909
Q3		
Non-Adjusted Model	1.035(0.782,1.370)	0.810
Minimally Adjusted Model	1.214(0.879,1.676)	0.238
Fully Adjusted Model	1.350(0.959,1.901)	0.085
Q4		
Non-Adjusted Model	1.473(1.127,1.926)	0.005
Minimally Adjusted Model	1.528(1.123,2.079)	0.007
Fully Adjusted Model	1.682(1.211,2.334)	0.002

Non-adjusted model adjusts for nothing. Minimally adjusted model adjusts for race/ethnicity, age, and PIR. Fully adjusted model adjusts for race/ethnicity, age, PIR, education, marital status, total cholesterol, HDL, glycohemoglobin, BMI, diabetes, hypertension, tumor history, smoke status, and drinking status.

Table S5. sensitivity analysis of the relationship between BPA and PSA after excluding tumor patients when PSA is a continuous variable

Exposure	Non-Adjusted Mode			Minimally Adjusted Model			Fully Adjusted Model		
	Estimate	95%CI	P -value	Estimate	95%CI	P-value	Estimate	95%CI	P -value
Urinary BPA	3.339	（1.665,5.012）	0.051	2.486	(0.943,4.029)	0.113	2.468	(0.972,3.964)	0.107
Urinary BPA									
Q1		Ref.			Ref.			Ref.	
Q2	-0.008	(-0.201,0.185)	0.996	0.090	(-0.081,0.261)	0.602	0.066	(-0.077,0.210)	0.646
Q3	0.080	(-0.105,0.264)	0.667	0.291	(0.103,0.478)	0.127	0.205	(0.054,0.356)	0.183
Q4	0.744	(0.465,1.024)	<0.001	0.631	(0.408,0.853)	<0.001	0.652	(0.461,0.843)	<0.001
p for trend			<0.001			<0.001			0.002

Non-adjusted model adjusts for nothing. Minimally adjusted model adjusts for race/ethnicity, age, and PIR. Fully adjusted model adjusts for race/ethnicity, age, PIR, education, marital status, total cholesterol, HDL, glycohemoglobin, BMI, diabetes, hypertension, tumor history, smoke status, and drinking status.

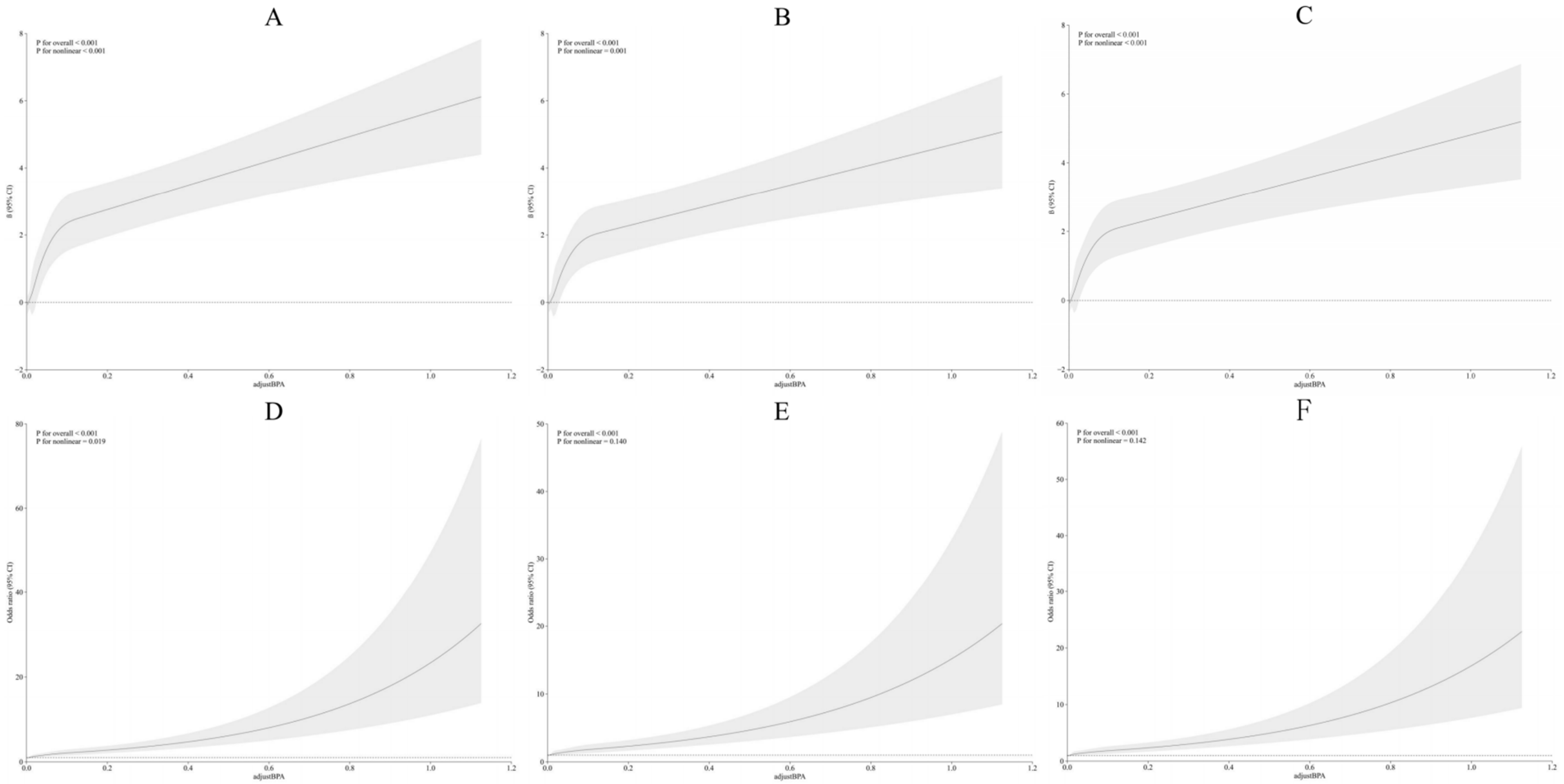

Figure S1. the non-linear relationship between BPA and PSA. PSA was included in the model as a continuous variable in A, B, and C. In D, E, and F, binary variables were included in the model.. Adjust BPA:Urine BPA concentration was estimated after adjustments for urine creatinine (BPA/Cr, ng/mg). A, D: Non-adjusted model adjusts for none. B, E: Minimally adjusted model adjusts for race/ethnicity, age, and PIR. C, F: Fully adjusted model adjusts for race/ethnicity, age, PIR, education, marital status, total cholesterol, HDL, glycohemoglobin, BMI, diabetes, hypertension, tumor history, smoke status, and drinking status.
